# Supplementary figures and images for: A deep learning approach for staging embryonic tissue isolates with small data
Source: PLoS One. 2021 Jan 8;16(1):e0244151. doi: 10.1371/journal.pone.0244151 (PMC7793293; doi:10.1371/journal.pone.0244151)

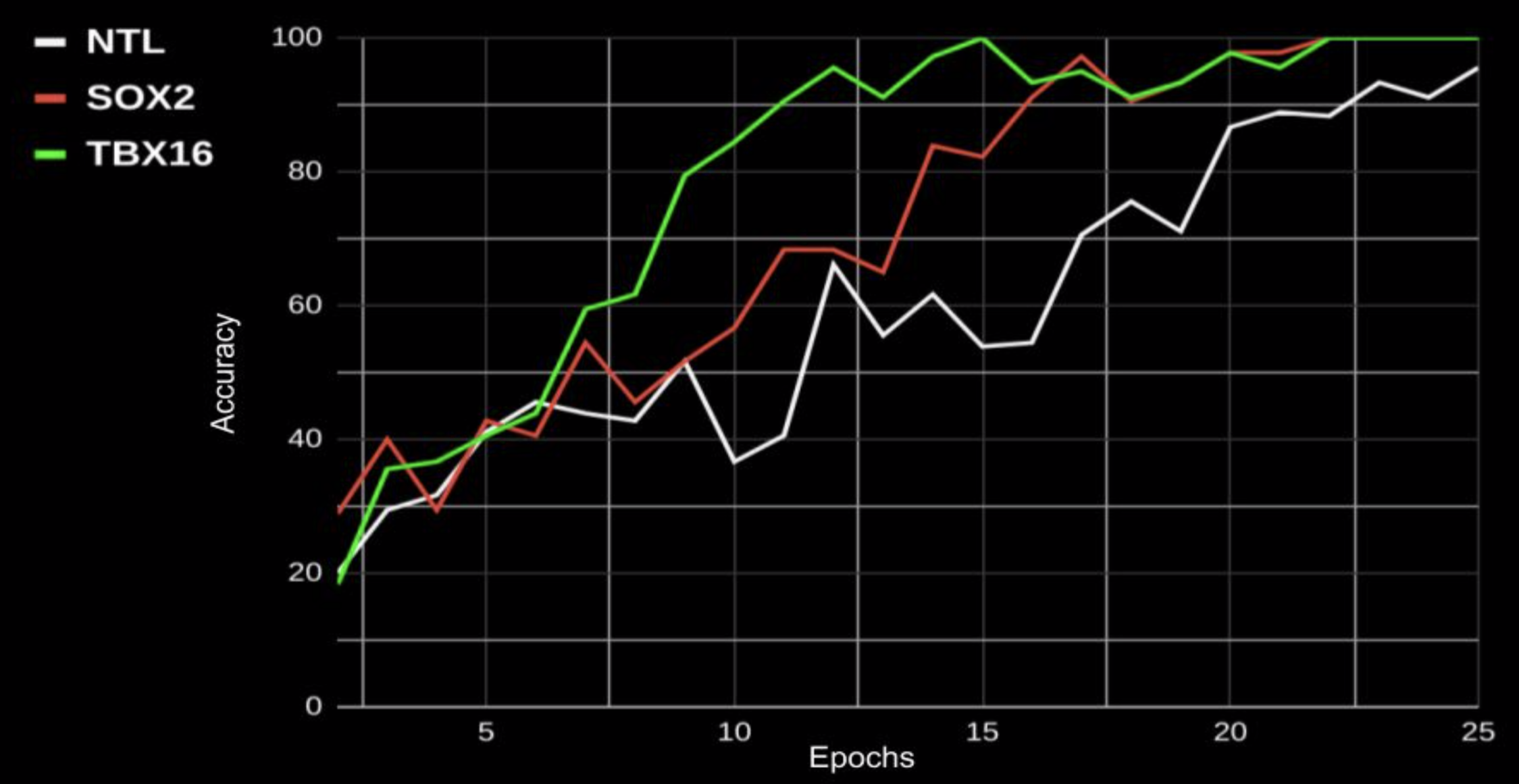

Supplement: S1 Fig — Accuracy is obtained at every epoch by averaging the accuracy scores for that particular epoch. (TIF) [file pone.0244151.s001.tif]

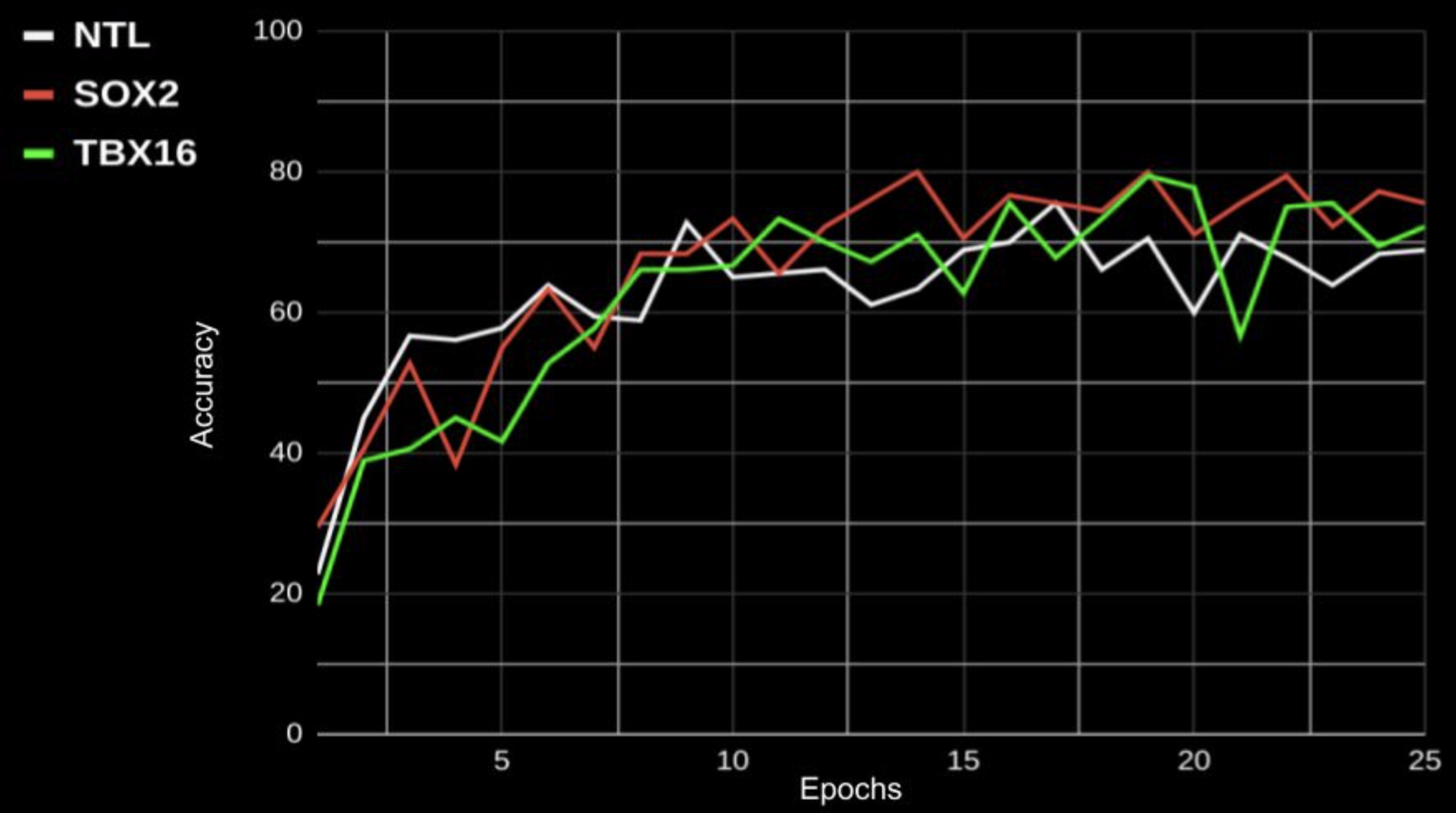

Supplement: S2 Fig — Accuracy is obtained at every epoch by averaging the accuracy scores for that particular epoch. (TIF) [file pone.0244151.s002.tif]
